# Effects of unsupervised campus HIIT on fitness and sleep in sedentary male students: a randomized controlled trial

**DOI:** 10.1186/s12889-026-27310-7

**Published:** 2026-04-09

**Authors:** Di Tang, Pengpeng Gou, Shunfang Liu, Xin Zhang, Danyang Li, Jinde Liu

**Affiliations:** 1 The Nethersole School of Nursing, Faculty of Medicine, The Chinese University of Hong Kong, Hong Kong, China; 2 Department of Sports Science and Physical Education, The Chinese University of Hong Kong, Hong Kong, China; 3 School of Sports Training, Wuhan Sports University, Wuhan, China; 4 Center of Strength and Conditioning, Wuhan Sports University, Wuhan, China; 5 School of Physical Education and Sports Science, South China Normal University, Guangzhou, China; 6 Faculty of Education, Central China Normal University, Hubei, China; 7 Faculty of Physical Education, Fudan University, Shanghai, China

**Keywords:** High-intensity interval training, Cardiorespiratory fitness, Sleep quality, University students, Campus-based intervention, VO_2max_, Pittsburgh Sleep Quality Index, Sedentary behavior

## Abstract

**Background:**

Sedentary behavior and poor sleep quality are prevalent among university students and are associated with reduced cardiorespiratory fitness. This randomized controlled trial evaluated the effects of a six-week, campus-based running high-intensity interval training (HIIT) program on physical fitness, sleep quality, and body composition in sedentary male university students.

**Methods:**

Fifty-six participants (age 21.6 ± 2.4 years) completed the study and were analyzed (HIIT group, *n* = 28; control group, *n* = 28). The unsupervised six-week intervention consisted of three weekly sessions of repeated 1-min high-intensity running bouts with recovery periods. Physical fitness, sleep quality, and body composition were assessed. Within- and between-group comparisons were conducted using t-tests, the Wilcoxon signed-rank test, or the Mann–Whitney U test, depending on data normality. Group-by-time interactions were examined using generalized estimating equations.

**Results:**

Significant group-by-time interactions were observed for VO_2max_ (*β* = − 6.63, 95% CI − 8.77 to − 4.49, *p* < 0.001, d = 1.019) and balance (*β* = − 23.11, 95% CI − 41.16 to − 5.06, *p* = 0.012, d = 0.544), with the HIIT group showing significant improvements (19.24% and 49.64%, respectively), whereas controls did not. While the group-by-time interaction for sleep quality was not significant, a favorable trend toward within-group improvement was observed in the HIIT group (− 0.57 points, − 12.15%, *p* = 0.082, d = 0.270), whereas no such trend occurred in the control group (0.07 points, 1.28%, *p* = 0.892, d = 0.029). No significant changes were observed for the remaining physical fitness indicators or body composition.

**Conclusions:**

A six-week, unsupervised campus-based HIIT running program improved cardiorespiratory fitness and balance while showing good adherence and safety in sedentary male university students, confirming its feasibility in a real-world setting. Potential benefits for sleep quality warrant validation in larger randomized trials.

**Trial registration:**

ClinicalTrials.gov (NCT07256847).

**Supplementary Information:**

The online version contains supplementary material available at 10.1186/s12889-026-27310-7.

## Introduction

In contemporary university settings, sedentary behavior and sleep disturbances have become prevalent and tightly interwoven lifestyle characteristics among students [1]. Driven by intense academic competition, students normalize prolonged sitting for study [2, 3], while widespread electronic media use extends screen exposure and fuels bedtime procrastination [4, 5]. This lifestyle pattern places students under dual pressures of “extreme physical inactivity” and “sleep deprivation” [6, 7]. Such excessive sedentary time, often accompanied by irregular routines and blue-light exposure, disrupts circadian rhythms and impairs sleep onset, duration, and quality [8–10], significantly compromising students’ physical and psychological well-being [4, 11].

Within this dual-pressure lifestyle, physical inactivity drives a comprehensive decline in physical fitness [12]. The primary manifestation of this inactivity is a direct reduction in cardiorespiratory fitness (CRF, typically indicated by VO_2max_) [13]. As a highly sensitive indicator of an inactive state, a reduced VO_2max_ signals diminished metabolic efficiency, lower fatigue resistance, and compromised functional capacity in daily activities [14–16]. Without sufficient physical exertion, the body’s adaptive capacities and overall physiological functioning are significantly impaired [17, 18].

Crucially, prolonged sedentary behavior and sleep problems exhibit a bidirectional negative interaction that further exacerbates these health risks [12]. On one hand, excessive sitting, especially when coupled with the aforementioned lack of physical exertion, results in extremely low daily energy expenditure [19]. This prevents the accumulation of sufficient “sleep pressure”, thereby hindering deep, restorative sleep [20]. On the other hand, poor sleep subsequently suppresses growth hormone secretion and parasympathetic recovery [21, 22]. This lack of recovery depletes physical reserves and diminishes the motivation to exercise, which in turn reinforces sedentary habits [19]. Ultimately, this vicious cycle escalates the risks for cardiovascular disease, metabolic syndrome (often accompanied by adverse changes in body composition, such as increased fat accumulation and reduced skeletal muscle mass), and cognitive decline, highlighting the urgent need for interventions that simultaneously target both physical fitness and sleep quality [23, 24].

For time-constrained university students, high-intensity interval training (HIIT) might be an ideal strategy to improve physical fitness due to its high time efficiency [25, 26]. Through brief bouts of high-intensity exertion, HIIT can induce substantial CRF-related adaptations and, in theory, may also improve sleep architecture by modulating core body temperature and hormonal profiles (such as cortisol–melatonin balance) [27, 28]. Compared to low-to-moderate-intensity continuous exercise that requires a longer duration, HIIT is more time-efficient, typically requiring only 10–30 min per session [29]. This feature aligns closely with the realities of university life, characterized by limited discretionary time, heavy academic workloads, and fast-paced schedules, making it easier to implement during short breaks throughout the day [30]. Despite these potential benefits, several research gaps need to be addressed to establish its effectiveness in university settings.

Two major limitations exist in the current literature. First, in terms of experimental design, although some HIIT studies include sleep-related measures, research specifically targeting sedentary university students is extremely limited, resulting in a lack of systematic evidence on sleep changes within this population [31]. Furthermore, most available studies focus predominantly on female participants. Only two studies have investigated male students [32, 33]. Given that sex differences may influence training adaptability, physiological responses, and physical fitness–related outcomes, this single-sex sampling approach introduces potential confounding and reduces the clarity and consistency of conclusions regarding HIIT in this population [34, 35].

Second, most previous studies have implemented HIIT through laboratory-based equipment (e.g., cycle ergometers or treadmills) or standardized protocols requiring professional supervision, which have limited ecological validity in campus settings [36, 37]. Consequently, there is a complete lack of unsupervised, campus-based HIIT research. To address these logistical barriers and improve ecological validity, running-based HIIT presents a superior alternative. Unlike equipment-dependent modalities, running requires minimal facilities and incurs no costs, making it highly accessible for students [38]. Physiologically, it effectively engages large muscle groups and provides substantial metabolic and cardiovascular stimuli, making it particularly effective for improving maximal oxygen uptake [39]. Moreover, the self-paced nature of running allows participants to monitor intensity without professional supervision, ensuring both safety and feasibility [40]. This accessibility makes running-based HIIT particularly well-suited for integration into the daily routines of university students, yet its specific impact on physical fitness and sleep quality in this demographic remains under-investigated.

Against this background, by eliminating reliance on laboratory equipment and adopting a track-based running protocol, this study sought to enhance the practical value and applicability of its findings. Through empirical evidence, we aimed to verify whether campus-based HIIT can serve as a feasible and effective comprehensive health intervention to disrupt the vicious cycle of sedentary behavior and sleep disorders. Based on previous related studies, we hypothesized that a six-week HIIT intervention would improve physical fitness and sleep quality (reflected by improvements in physical fitness test scores and a reduction in Pittsburgh Sleep Quality Index scores, respectively). Overall, the primary aim of the present study was to investigate the effects of an unsupervised, campus-based running HIIT program on physical fitness, sleep quality, and body composition in sedentary male university students.

## Methods

### Participants

Participants were recruited through multiple university channels, including the institution’s mass email system and official social media platforms such as WeChat groups and Facebook communities. Interested individuals completed an online screening questionnaire designed to evaluate eligibility based on predefined inclusion and exclusion criteria. Sedentary behavior status was determined using the sitting-time item from the International Physical Activity Questionnaire–Short Form (IPAQ-SF) [41, 42]. In accordance with guidelines from the Sedentary Behaviour Research Network (SBRN) and commonly used thresholds in university-based studies, individuals reporting ≥ 8 h of sitting per day were classified as sedentary and deemed eligible for further assessment [43]. To avoid the confounding influence of pre-existing exercise habits on the intervention outcomes, only candidates engaging in fewer than two structured exercise sessions per week were included [44]. Detailed inclusion and exclusion criteria are summarized in Table 1.

**Table 1:** Eligibility criteria for the target participant

Inclusion criteria	Exclusion criteria
Male university students	Neurological, psychiatric, cardiovascular, or musculoskeletal disorders
Sedentary behavior (≥ 8 h/day sitting, IPAQ-SF)	Hypertension or heart disease
Engaging in < 2 structured exercise sessions per week	Other medical conditions that increase exercise risk
Able to understand Chinese or English	Use of medications affecting cardiovascular responses or exercise tolerance
Cleared for exercise participation (PAR-Q)	

*IPAQ-SF* International Physical Activity Questionnaire–Short Form, *PAR-Q* Physical Activity Readiness Questionnaire

To estimate the minimum number of participants required for adequate statistical power, an a priori power analysis was conducted using G*Power (version 3.1.9.7). The calculation was based on a two-group repeated-measures design (group × time), assuming an alpha level of 0.05, a statistical power of 0.80, and a small-to-moderate expected effect size (*f* = 0.20), consistent with previous HIIT interventions in young adults [45]. The analysis indicated that a minimum of 52 participants would be required. To account for a potential 10% attrition rate commonly reported in exercise-intervention studies, the planned recruitment target was set at approximately 60 participants.

### Experimental design

The protocol of this study was prospectively registered in the ClinicalTrials.gov registry (NCT07256847). Participants were randomly assigned to the HIIT or control group using a computer-generated sequence administered by an independent researcher to ensure allocation concealment.

**Fig. 1:**
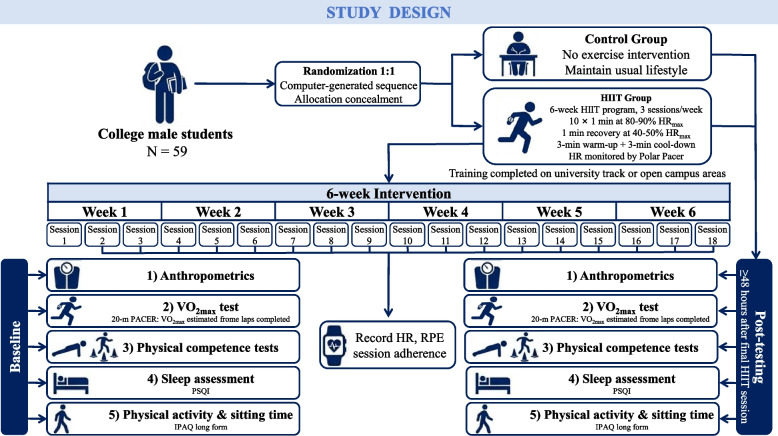
Study design of the randomized controlled trial. Overview of participant recruitment, randomization, six-week intervention period, and pre- and post-intervention outcome assessments (final analysis *n* = 56; 28 per group)

**Fig. 2:**
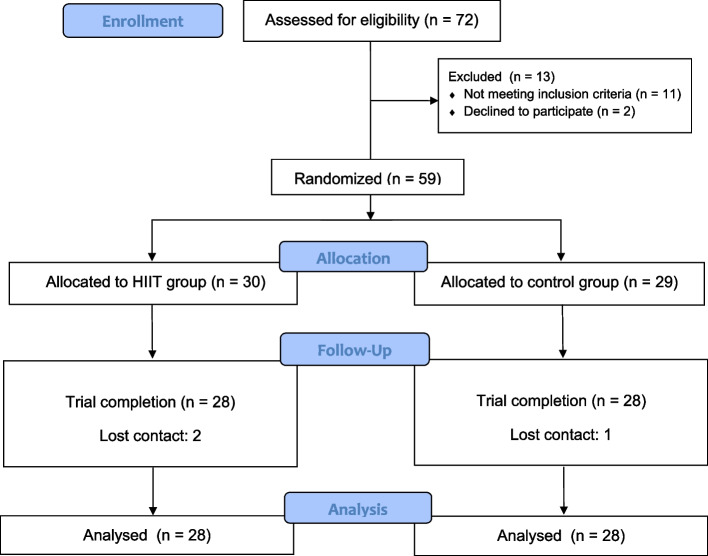
Flow chart of the experimental procedure. Flow of participants from initial screening (*n* = 72) to final analysis (*n* = 56; HIIT = 28; control = 28)

Following online and on-site recruitment procedures, 59 volunteers met the eligibility criteria and were invited to participate. To minimize measurement bias, all assessments relying on instrumentation were conducted by trained evaluators blinded to group allocation. Participants assigned to the intervention group completed a six-week HIIT program, while those in the control group did not receive any exercise intervention during the study period. Baseline assessments were conducted prior to randomization, and post-intervention assessments were completed at the end of the sixth week. Ultimately, of the 59 enrolled participants, 56 completed the study and were included in the final analysis (28 per group). The experimental procedure is shown in Figs. 1 and 2.

This study was reported in accordance with the CONSORT 2010 guidelines, and the completed checklist is available in the Supplementary Material A.

### Measurement

#### Physical fitness

To avoid fatigue-related interference, physical fitness assessments were divided into two separate testing sessions spaced at least 48 h apart [46].

In the first session, CRF was assessed using the 20-m Progressive Aerobic Cardiovascular Endurance Run (PACER) test, a validated field-based measure widely used to estimate VO_2max_ (measured in mL·kg⁻^1^·min⁻^1^) in youth and young adults [47]. Briefly, participants ran back and forth across a 20-m distance, pacing themselves to pre-recorded audio signals (beeps). The required running speed progressively increased at regular intervals throughout the test. The test was terminated when a participant failed to reach the 20-m line before the beep on two consecutive occasions or reached volitional exhaustion. Although field tests inherently introduce measurement error, prior work demonstrates that the PACER test yields error margins within acceptable limits [48]. VO_2max_ was estimated from the total laps completed using the Léger et al. equation, a widely validated method for young populations [47, 49].

In the second session, other components of physical fitness were assessed using standardized field-based tests. Core endurance was evaluated using the plank test, with time to failure used as the outcome (recorded in seconds) [50]. Participants maintained a prone plank position with forearms and toes in contact with the ground, keeping the body in a straight line until volitional exhaustion or loss of proper form. Static balance was assessed using the unipedal stance test [51], during which participants stood barefoot on one leg with hands on their hips and eyes closed, and the duration of maintaining balance was recorded (in seconds). Agility was measured with the Agility T-test following established procedures [52], requiring participants to sprint forward, shuffle laterally, and backpedal between cones arranged in a T-shape, with total completion time recorded (in seconds). Flexibility was evaluated with the Sit-and-Reach test [53], in which participants reached forward along a standardized box while seated with legs fully extended, and the maximal reach distance was recorded (in centimeters).

#### Sleep quality

Participants were instructed to maintain their usual sleep patterns throughout the six-week intervention. Sleep quality was measured using the Pittsburgh Sleep Quality Index (PSQI) [54], administered at baseline and post-intervention. The PSQI yields seven component scores and a global score (ranging from 0 to 21 points), with higher values indicating poorer sleep.

#### Body composition

Body composition was evaluated using a multi-frequency bioelectrical impedance analyzer (MC-780MA, Tanita Corporation, Tokyo, Japan) [55]. Participants stood barefoot on the platform with full contact between feet and electrodes while holding the hand electrodes with correct thumb–finger placement. They remained still for approximately one minute with extended elbows and shoulders abducted to approximately 30°. The specific parameters extracted from the analyzer included body weight (in kg), body mass index (BMI, in kg/m^2^), body fat (in kg), body fat percentage (%), total muscle mass (in kg), upper limb muscle mass (in kg), and lower limb muscle mass (in kg). Additionally, waist circumference (in cm) and hip circumference (in cm) were measured using a standard non-elastic measuring tape. Participants adhered to a minimum three-hour fasting period and refrained from exercise on the day of measurement. Although BIA recommendations suggest an eight-hour fasting window [55, 56], this could not be consistently implemented due to scheduling constraints. Therefore, a three-hour protocol was adopted, as this duration is considered adequate to mitigate the acute effects of recent food and fluid intake on impedance, consistent with previous research [57, 58].

#### Physical activity and sitting time

The evaluation of physical activity levels was conducted using the self-administered International Physical Activity Questionnaire (IPAQ) long form [41]. This version of the IPAQ consists of 27 items designed to assess the duration (in minutes) of participants’ engagement in physical activities across different intensity domains. The primary outcomes derived from the IPAQ were total physical activity (expressed in MET-min/week) and average daily sitting time (in minutes/day).

To prevent potential confounding effects arising from unplanned or habitual aerobic exercise during the six-week intervention period, individuals with an established regular exercise routine were excluded during recruitment. This measure ensured that the observed intervention effects could be more accurately attributed to the study protocol rather than pre-existing physical activity behaviors.

In addition, to minimize the confounding effects of dietary changes, participants were asked to maintain their usual eating habits throughout the six-week intervention period. The study used a 6-day food journal to monitor participants’ dietary intake, collected at two time points: the three days before and the three days after the intervention. Each monitoring period consisted of two weekdays and one weekend day. Participants used photographs to record all their dietary intake. Based on these records, the estimated daily caloric intake (in kcal) and macronutrient intake (carbohydrates, proteins, and fats in grams) were calculated to verify that participants maintained consistent eating patterns throughout the intervention.

### Intervention

The intervention protocol was informed by evidence suggesting that HIIT programs lasting over four weeks produce meaningful physiological adaptations [59] and by ACSM and prior research recommending 6–12-week programs conducted 3–7 times per week [60, 61]. Accordingly, a six-week HIIT program comprising three sessions per week was implemented. Participants performed sessions during available breaks in their daily schedule using the university track or nearby open spaces. All participants completed the Physical Activity Readiness Questionnaire (PAR-Q) before participation to ensure exercise readiness.

Based on prior research demonstrating its efficacy, the HIIT protocol consisted of 10 × 1-min intervals at 80–90% HR_max_, interspersed with 1-min recovery periods at 40–50% HR_max_ [62–64]. HR_max_ was estimated using the age-predicted formula *HR*_*max*_ = *208 − 0.7* × *age*, which has demonstrated accuracy in populations similar to the present sample [65, 66]. This protocol has shown meaningful short-term effects on VO_2max_ [67, 68], and was deemed appropriate for insufficiently active male students. Each session included a standardized 3-min warm-up and 3-min cool-down, with an average total duration of 26 min. Exercise intensity was monitored continuously using Polar Pacer heart rate monitors. Participants also provided ratings of perceived exertion (RPE) using the Borg 6–20 scale [69], aiming for RPE 15–18 during high-intensity bouts and RPE 11–13 during low-intensity periods [70]. Sessions were initially supervised by qualified trainers for safety and familiarization. Once participants demonstrated correct execution and could meet intensity requirements independently, sessions were completed unsupervised on campus. Polar Flow for Coach was used, with participant consent, to verify adherence and intensity. To verify exercise intensity using Polar Flow data, an individual training session was classified as intensity-compliant if the participant reached the target zone of ≥ 80% HR_max_ during at least 80% of the high-intensity intervals.

Participants were allowed to undertake the training either on a treadmill or outdoors, through running, jogging, and brisk walking, provided they reached the stipulated heart rate. During the initial three sessions, participants were obliged to exercise in a laboratory under the guidance and supervision of research staff. This ensures not only a precise understanding and execution of the training regimen but also the safety of participants during subsequent unsupervised exercises. Once it was ascertained that participants could meet the training benchmarks without supervision, participants continued the remaining sessions within the campus environment, scheduling their three weekly workouts in accordance with their regular on-campus routines. HIIT has also been demonstrated to be implemented safely and successfully in a campus-based environment [70]. During these campus-based exercises, participants were required to wear a Polar Pacer to track exercise intensity. The monitor records the exercise data throughout the training program, and with prior consent obtained from participants, we monitored each participant’s exercise data through the Polar Flow for Coach, including metrics such as heart rate, frequency, and running speed to confirm participants’ compliance with the exercise plan. This campus-based arrangement maximizes the use of the university environment to increase physical activity despite academic and electronic-media pressures, thereby reducing sedentary behavior and establishing a sustainable intervention model for real campus settings rather than laboratory environments, and the methodological approach has also been employed in prior studies [71, 72].

### Statistical analysis

All statistical analyses were performed using IBM SPSS Statistics (Version 29.0, IBM Corp., Armonk, NY, USA). To evaluate the true physiological efficacy of the intervention under optimal compliance, the primary analyses were conducted based on the per-protocol (PP) population, which included all participants who successfully completed the intervention and both baseline and post-intervention assessments [73]. Continuous variables were presented as means and standard deviations. The normality of each variable was assessed using the Shapiro–Wilk test, and the results informed the selection of subsequent statistical procedures. For variables that met the normality assumption, paired t-tests were used to evaluate within-group changes, and independent-samples t-tests were used to compare between-group differences at baseline and post-intervention. For variables that were not normally distributed, non-parametric tests (the Wilcoxon signed-rank test for within-group comparisons and the Mann–Whitney U test for between-group comparisons) were applied. Additionally, effect sizes were calculated (Cohen’s d) to evaluate the magnitude of the intervention effects, with values of 0.2, 0.5, and 0.8 considered small, medium, and large, respectively. Statistical significance was set at *p* < 0.05.

To examine the overall intervention effects, a Generalized Estimating Equation (GEE) model was employed to evaluate group-by-time interactions. The GEE approach was selected as it is highly robust to non-normal data distributions (as observed in some of our variables) and effectively handles correlated repeated measures within subjects without requiring strict distributional assumptions. Compared to traditional repeated-measures analysis of variance (ANOVA), GEE is highly robust to non-normal data distributions (as observed in some of our variables) and effectively handles correlated repeated measures within subjects without requiring strict distributional assumptions. Furthermore, when compared to alternative approaches such as generalized linear mixed models (GLMMs), the GEE model was specifically chosen because our primary objective was to evaluate the population-average (marginal) effects of the HIIT intervention on the sample, rather than focusing on subject-specific individual trajectories. Finally, GEE utilizes robust (empirical) standard errors, which provide valid statistical inferences even if the within-subject working correlation structure is slightly misspecified, thereby ensuring a more conservative and reliable estimation for our study design. For the continuous outcomes reported in this study, a Gaussian distribution with an identity link function was specified. An exchangeable working correlation structure was selected to account for within-subject correlations across repeated measurements (detailed Intraclass Correlation Coefficients for each outcome are provided in Supplementary Material B). Furthermore, robust standard errors were applied to ensure valid statistical inferences regardless of the specified correlation structure or potential minor violations of distributional assumptions. Age was included as a predetermined covariate. Additionally, if daily dietary intake showed significant changes from baseline during the intervention period, these nutritional variables were incorporated as covariates in the final analysis to account for the potential influence of dietary variations on physiological outcomes. When a significant group-by-time interaction was observed, subsequent pairwise comparisons were conducted to explore the specific within-group and between-group differences.

To assess the robustness of our primary findings and account for participant attrition (*n* = 3), a sensitivity analysis was performed based on the intention-to-treat (ITT) principle. Missing post-intervention data for the dropouts were handled using the last observation carried forward (LOCF) method, which provides a conservative estimate by assuming no change from baseline [73]. The aforementioned statistical procedures were re-analyzed on this full dataset to verify the consistency of the intervention effects and ensure that the dropouts did not bias the primary outcomes.

## Results

The primary analyses of this randomized controlled trial revealed significant group-by-time interactions for VO_2max_ (*β* = − 6.63, 95% CI − 8.77 to − 4.49, *p* < 0.001, d = 1.019) and balance (*β* = − 23.11, 95% CI − 41.16 to − 5.06, *p* = 0.012, d = 0.544) following the six-week HIIT intervention. Additionally, while sleep quality showed a trend toward improvement (decreased by 0.57 points, 12.15%; *p* = 0.082), it did not reach statistical significance, and no significant interactions were observed for other physical fitness and body composition outcomes.

### Characteristics of the participants

The mean age of the participants included in the final analysis was 21.6 ± 2.4 years, with comparable values in the HIIT group (21.8 ± 2.4 years) and control group (21.5 ± 2.4 years). The two groups did not differ significantly at baseline in body weight, BMI, or other anthropometric measures (Table 2). Nutritional variables (protein, fat, carbohydrates, and total energy intake) showed no significant group–time interactions (all *p* > 0.05), but protein intake differed significantly between groups at baseline (*p* = 0.007). As participants were randomly assigned, this baseline difference is considered an unexpected chance finding. To ensure statistical rigor, a post-hoc decision was made to adjust for this baseline difference. Therefore, baseline protein intake, along with the predetermined covariate of age, was incorporated into the subsequent GEE repeated-measures models.

**Table 2:** Participant characteristics and nutritional intake

Parameter	Time point	HIIT group (*n* = 28)	Control group (*n* = 28)	*p*
Age	Pre	21.79 (2.38)	21.46 (2.41)	0.539
Height	Pre	174.71 (6.57)	175.02 (5.74)	0.850
Weight	Pre	68.60 (8.79)	72.10 (13.68)	0.261
BMI	Pre	22.49 (2.91)	23.53 (4.23)	0.291
Protein	Pre	66.22 (2.84)	68.41 (4.35)	0.007
	Post	66.16 (5.08)	68.39 (4.12)	0.880
Fat	Pre	56.72 (3.65)	57.87 (5.34)	0.256
	Post	58.15 (3.49)	57.16 (5.26)	0.503
Carbohydrate	Pre	235.06 (6.87)	235.44 (8.48)	0.447
	Post	235.87 (7.59)	237.81 (6.20)	0.174
Energy intake	Pre	1715.63 (45.38)	1738.22 (48.77)	0.083
	Post	1731.58 (39.74)	1739.41 (45.43)	0.686

Values are presented as mean ± SD

Units are as follows: age (years), height (cm), weight (kg), BMI (kg/m^2^), protein/fat/carbohydrate (g/day), energy intake (kcal/day)

*HIIT* high-intensity interval training, *BMI* body mass index

### Intervention adherence and safety

Among those who completed the intervention, attendance at the prescribed training sessions was 100%, and the overall intensity compliance rate was 86.51%. Detailed session-by-session attendance and intensity compliance rates are provided in Supplementary Material C. No adverse events were reported.

### Physical fitness

**Fig. 3:**
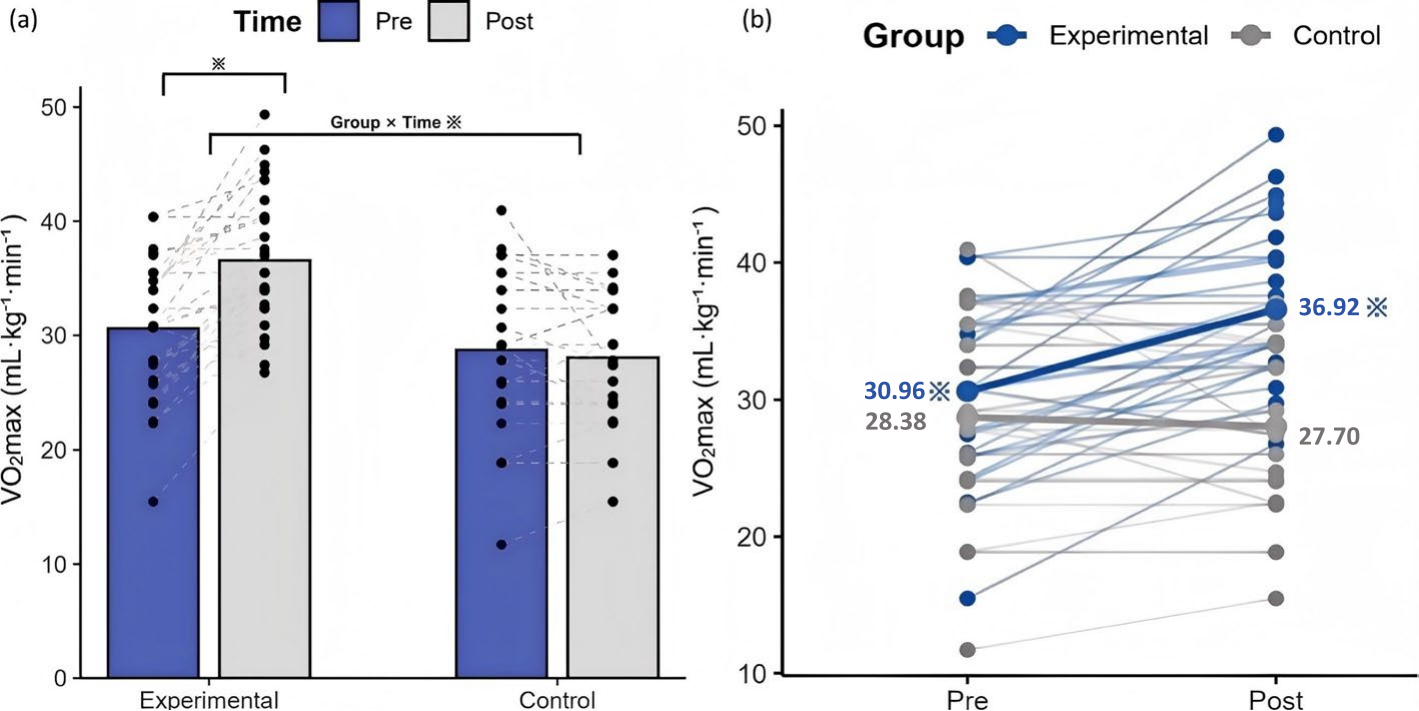
Changes in VO_2max_ from pre- to post-intervention in the HIIT and control groups. **a** The bar chart represents the estimated marginal means (adjusted for age and pre-intervention protein intake). Blue bars indicate pre-intervention values and grey bars indicate post-intervention values. Individual participant raw data are shown as black dots, with dashed lines connecting paired pre–post measurements. **b** Individual trajectories (raw values) across time. Blue lines represent participants in the HIIT group and grey lines represent participants in the control group. Thick lines indicate the estimated marginal group means. * *p* < 0.05

A significant group-by-time interaction was observed in VO_2max_ (*β* = − 6.63, 95% CI: − 8.77 to − 4.49, *p* < 0.001; Table 3), indicating a significantly greater improvement in the HIIT group compared to the control group. Post-hoc tests indicated that the HIIT group experienced a significant improvement in VO_2max_ following the intervention, increasing by 5.96 mL·kg⁻^1^·min⁻^1^ in absolute terms and by 19.24% relative to baseline (*p* < 0.001), while the control group remained relatively stable*,* showing a decrease of 0.67 mL·kg⁻^1^·min⁻^1^ and 2.36% (*p* = 0.298; Fig. 3). Furthermore, at post-intervention, VO_2max_ was significantly higher in the HIIT group than in the control group (*p* < 0.001).

**Table 3:** Effects of HIIT on physical fitness and sleep quality

Outcome	HIIT group (*n* = 28)	Control group (*n* = 28)	*p*		Group × Time		
					***β* (95% CI)**^**a**^	***p***	***ES***^***b***^
PACER							
Pre	39.96 (13.68)	34.21 (14.03)	0.126 (t = 1.553)		− 17.93 (− 23.92, − 11.94)	<.001	1.294
Post	56.75 (16.54)	33.07 (11.80)	<.001 (Z = − 4.902)				
*p*	<.001 (t = − 6.689)	0.760 (Z = − 0.305)					
VO_2max_							
Pre	30.61 (6.01)	28.72 (6.97)	0.281 (t = 1.088)		− 6.63 (− 8.77, − 4.49)	<.001	1.019
Post	36.57 (5.81)	28.05 (5.85)	<.001 (t = 5.469)				
*p*	<.001 (t = − 6.626)	0.316 (t = 1.022)					
Balance							
Pre	38.46 (25.53)	60.38 (54.38)	0.176 (Z = − 1.352)		− 23.11 (− 41.16, − 5.06)	0.012	0.544
Post	57.73 (40.14)	56.54 (55.93)	0.367 (Z = − 0.901)				
*p*	0.004 (Z = − 2.869)	0.716 (Z = 0.364)					
Agility							
Pre	14.87 (2.05)	15.34 (1.94)	0.451 (Z = − 0.754)		− 0.80 (− 2.35, 0.74)	0.307	0.401
Post	14.71 (3.67)	14.38 (2.83)	0.342 (Z = − 0.951)				
*p*	0.031 (Z = − 2.152)	0.015 (Z = − 2.437)					
Flexibility							
Pre	24.46 (11.51)	23.40 (10.24)	0.719 (t = 0.362)		− 1.50 (− 4.93, 1.94)	0.393	0.137
Post	26.30 (10.34)	23.75 (12.78)	0.415 (t = 0.821)				
*p*	0.254 (t = − 1.166)	0.674 (t = − 0.425)					
Core endurance							
Pre	120.84 (52.04)	104.55 (41.04)		0.199 (t = 1.300)	− 11.89 (− 30.45, 6.66)	0.209	0.254
Post	132.67 (65.64)	104.49 (46.39)		0.070 (t = 1.855)			
*p*	0.165 (t = − 1.428)	0.838 (Z = − 0.205)					
Sleep							
Pre	4.75 (2.11)	5.43 (2.44)		0.272 (t = − 1.111)	0.64 (− 0.57, 1.86)	0.299	0.281
Post	4.18 (1.85)	5.50 (1.77)		0.009 (t = − 2.730)			
*p*	0.100 (t = 1.706)	0.895 (t = − 0.134)					

Values are presented as mean ± SD

Units are as follows: PACER (laps), VO_2max_ (mL·kg^−1^·min^−1^), balance (s), agility (s), flexibility (cm) and, core endurance (s)

^a^Adjusted for age and pre-intervention protein intake

^b^Effect sizes were reported as the absolute values of Cohen’s d

A significant group–time interaction was also observed in balance (*β* = − 23.11, 95% CI − 41.16 to − 5.06, *p* = 0.012; Table 3). Post-hoc tests indicated that balance improved in the HIIT group (19.27 s, 49.64%, *p* = 0.010), whereas no significant change was observed in the control group, which decreased by 3.84 s and by 6.39% (*p* = 0.478). At post-intervention, no significant difference was observed between groups (*p* = 0.926). No significant group–time interactions were detected for the other outcome measures, including agility, flexibility, and core endurance.

### Sleep quality

**Fig. 4:**
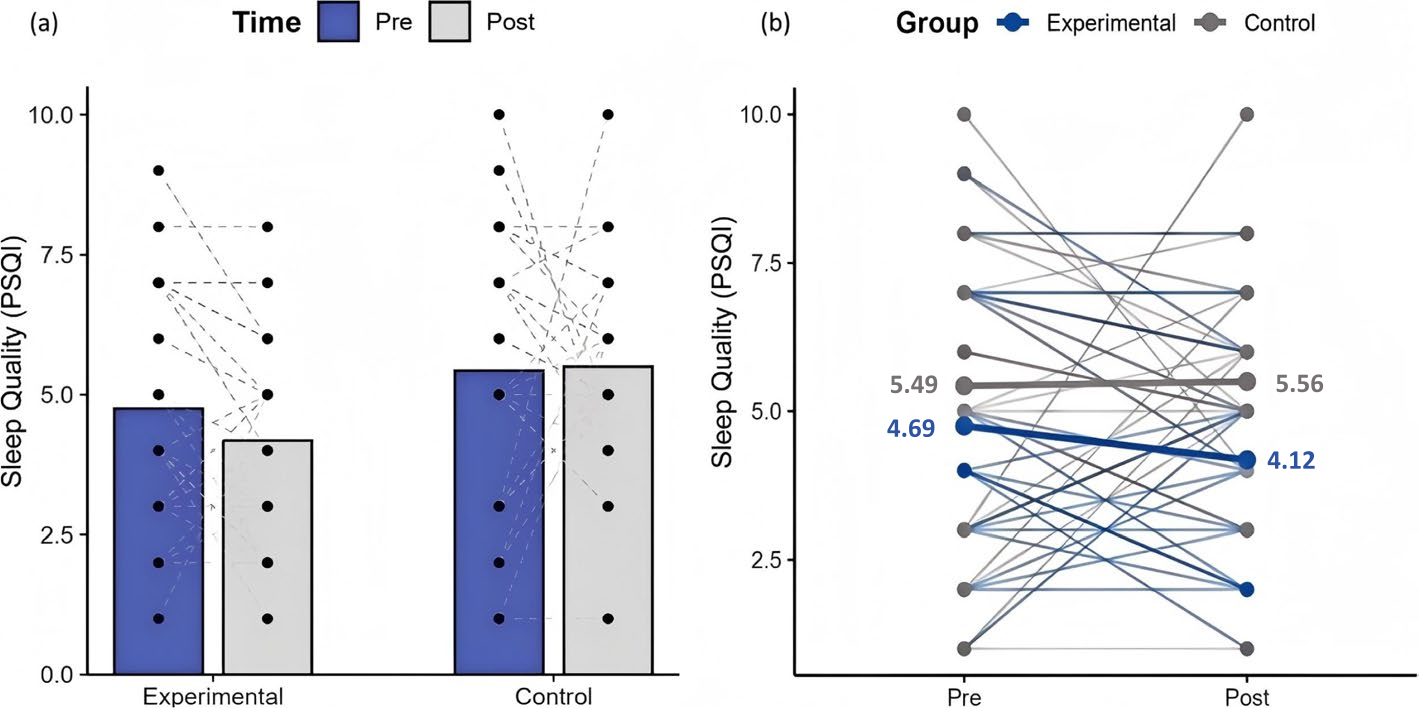
Changes in sleep quality from pre- to post-intervention in the HIIT and control groups. **a** The bar chart represents the estimated marginal means (adjusted for age and pre-intervention protein intake). Blue bars indicate pre-intervention values and grey bars indicate post-intervention values. Individual participant raw data are shown as black dots, with dashed lines connecting paired pre–post measurements. **b** Individual trajectories (raw values) across time. Blue lines represent participants in the HIIT group and grey lines represent participants in the control group. Thick lines indicate the estimated marginal group means. Higher PSQI scores indicate poorer sleep quality

While the overall group–time interaction for sleep quality did not reach statistical significance (*β* = 0.64, 95% CI − 0.57 to 1.86, *p* = 0.299; Table 3), post-hoc analyses based on the adjusted model suggested a favorable trend in the HIIT group. Specifically, sleep quality scores in the HIIT group decreased by 0.57 points (12.15%; *p* = 0.082). In contrast, no such trend was observed in the control group, which showed a slight increase of 0.07 points (1.28%; *p* = 0.892; Fig. 4).

### Body composition and physical activity level

No significant group–time interaction was detected for any of the other outcome measures (weight, body fat, body fat percentage, muscle mass, BMI, waist circumference, hip circumference, and physical activity level) (Table 4).

**Table 4:** Effects of HIIT on body composition and physical activity

Outcome	HIIT group (*n* = 28)	Control group (*n* = 28)	*p*	Group × Time		
				***β* (95% CI)**^**a**^	***p***	***ES***^***b***^
Weight						
Pre	68.60 (8.79)	72.10 (13.68)	0.261 (t = − 1.137)	− 0.65 (− 2.56, 1.27)	0.507	0.057
Post	69.15 (8.95)	72.00 (13.62)	0.360 (t = − 0.924)			
*p*	0.571 (t = − 0.574)	0.776 (Z = − 0.285)				
Body fat						
Pre	14.15 (5.61)	16.64 (9.48)	0.512 (Z = − 0.656)	− 0.43 (− 1.78, 0.92)	0.533	0.056
Post	14.15 (5.77)	16.20 (9.05)	0.611 (Z = − 0.508)			
*p*	0.992 (t = 0.011)	0.053 (Z = − 1.937)				
Body fat percentage						
Pre	0.20 (0.06)	0.22 (0.08)	0.412 (t = − 0.827)	− 0.03 (− 0.18, 0.12)	0.701	0.141
Post	0.20 (0.06)	0.21 (0.08)	0.485 (t = − 0.704)			
*p*	0.775 (t = 0.289)	0.046 (t = 2.093)				
Muscle mass						
Pre	51.00 (6.09)	52.45 (4.92)	0.329 (t = − 0.985)	− 0.66 (− 2.20, 0.88)	0.401	0.117
Post	52.14 (5.58)	52.94 (5.26)	0.585 (t = − 0.550)			
*p*	0.140 (t = − 1.521)	0.095 (t = − 1.731)				
Upper limb muscle						
Pre	4.88 (0.99)	5.00 (0.64)	0.589 (t = − 0.545)	− 0.05 (− 0.28, 0.19)	0.694	0.048
Post	4.95 (0.98)	5.03 (0.73)	0.747 (t = − 0.324)			
*p*	0.494 (t = − 0.694)	0.245 (Z = − 1.163)				
Lower limb muscle						
Pre	19.65 (2.86)	20.36 (2.17)	0.300 (t = − 1.046)	− 0.47 (− 1.19, 0.25)	0.199	0.185
Post	20.20 (2.65)	20.44 (2.01)	0.705 (t = − 0.380)			
*p*	0.130 (t = − 1.562)	0.499 (t = − 0.685)				
BMI						
Pre	22.49 (2.91)	23.53 (4.23)	0.291 (t = − 1.067)	− 0.20 (− 0.79, 0.39)	0.499	0.058
Post	22.66 (3.10)	23.49 (4.20)	0.403 (t = − 0.843)			
*p*	0.574 (t = − 0.568)	0.673 (t = 0.426)				
Waist circumference						
Pre	79.29 (9.90)	81.05 (10.30)	0.517 (t = − 0.652)	− 0.29 (− 2.53, 1.96)	0.803	0.029
Post	79.04 (7.87)	80.51 (10.71)	0.844 (Z = − 0.197)			
*p*	0.818 (t = 0.233)	0.201 (Z = − 1.278)				
Hip circumference						
Pre	96.96 (6.77)	98.06 (7.52)	0.566 (t = − 0.577)	1.54 (− 0.59, 3.67)	0.157	0.215
Post	95.93 (5.46)	98.57 (7.45)	0.136 (t = − 1.515)			
*p*	0.312 (t = 1.030)	0.223 (Z = − 1.219)				
Physical activity						
Pre	1953.68 (2053.27)	2442.95 (1869.39)	0.184 (Z = − 1.327)	− 552.48 (− 1472.84, 367.88)	0.239	0.281
Post	2324.77 (2091.58)	2261.57 (1712.82)	0.922 (Z = − 0.098)			
*p*	0.065 (Z = − 1.845)	0.464 (t = 0.743)				

Values are presented as mean ± SD

Units are as follows: weight (kg), body fat (kg), body fat percentage (%), muscle mass (kg), BMI (kg/m^2^), circumference (cm) and physical activity (MET-min/week)

^a^Adjusted for age and pre-intervention protein intake

^b^Effect sizes were reported as the absolute values of Cohen’s d

### Sensitivity analysis

The sensitivity analysis based on the ITT principle with LOCF for the three dropouts yielded results consistent with the primary per-protocol analysis, confirming that participant attrition (5.1%) did not bias the primary study outcomes. Detailed results of the ITT analysis are provided in Supplementary Material D.

## Discussion

The primary findings of this randomized controlled trial indicate that a six-week running-based HIIT program, delivered in a real-world university setting, significantly improved VO_2max_ and balance performance in sedentary male college students. Although the group-by-time interaction for sleep quality did not reach statistical significance, within-group improvements suggest a potential beneficial effect. Conversely, no significant changes were observed in body composition or other physical fitness indicators. Collectively, these results demonstrate the physiological efficacy and practical feasibility of implementing a low-resource HIIT protocol in a campus environment.

The significant improvement in VO_2max_ observed in this study aligns with established evidence regarding HIIT’s cardiorespiratory benefits [74–76]. Notably, the relative increase observed in the present study (19.24%) is higher than the average improvement of approximately 13% reported in previous meta-analyses of HIIT interventions. This greater magnitude of improvement may be attributable to the low baseline fitness level of the participants and the possible high responsiveness of sedentary individuals to training stimuli [68]. Crucially, this study confirms that a self-supervised, running-based protocol can elicit physiological adaptations comparable to laboratory-based interventions. From a physiological standpoint, repeated bouts of high-intensity interval running stimulate both cardiac pumping capacity and skeletal muscle oxygen utilization [77]. With continued training, adaptations may occur in stroke volume, cardiac output, mitochondrial function, and capillary density within peripheral tissues, collectively contributing to the observed elevation in VO_2max_ [77]. For sedentary students, this implies that a time-efficient regimen (requiring no technical skills or specialized facilities) can effectively counteract the physiological decline associated with inactivity, such as reduced metabolic efficiency [38]. This validates running-based HIIT as a scalable strategy to mitigate cardiovascular risks in resource-limited university settings.

Regarding sleep quality, although no significant group-by-time interaction was found, a trend toward pre-to-post improvement within the intervention group suggests HIIT may offer restorative benefits via autonomic and circadian regulation [78, 79]. The lack of a significant interaction likely also reflects methodological constraints. First, the training dose may have been insufficient. To maintain ecological validity within the academic semester, the intervention duration was shorter than protocols typically required to elicit robust sleep adaptations [80]. Second, the modest sample size may have limited the statistical power to detect a smaller effect size in sleep quality [81]. A post-hoc power analysis for the group-by-time interaction in sleep quality revealed a statistical power of less than 20%. Finally, screen time was not controlled. Given that nocturnal electronic device use is a potent sleep disruptor in students [8, 82], this unmeasured confounder may have obscured the specific effects of HIIT. Future studies should employ larger samples, longer durations, and screen-time monitoring to clarify these outcomes.

This study did not observe significant changes in body composition parameters. This likely results from two factors. First, participants had baseline weight and body fat within the normal range, which typically requires higher metabolic stimulation to alter [83]. Second, participants were instructed to maintain their daily dietary habits and did not receive any extra dietary restrictions. This may have limited the possibility of measurable changes [84]. Future research to explore the moderating effects of HIIT on body composition might consider combining HIIT with dietary control or resistance training to generate greater energy expenditure and stronger muscle metabolic stimulation, thereby increasing the likelihood of inducing meaningful adaptive changes in body composition.

In terms of other physical fitness outcomes, the HIIT group showed significant improvements in balance, suggesting that running-based training may induce foundational neuromuscular adaptations. The rapid support-swing transitions and center-of-mass adjustments inherent to high-intensity running likely stimulated lower-limb proprioception and core stability [85]. In contrast, no significant changes were detected in the remaining physical fitness measures, likely due to the specificity of the training stimulus. Running-based HIIT is primarily linear and cyclic, providing limited direct loading to agility, explosive strength, or core endurance [86]. The relatively short intervention period may also have been insufficient to elicit broader fitness adaptations [86].

Taken together, these findings validate the efficacy and feasibility of running-based HIIT in a campus setting. Conducted without laboratory facilities or direct supervision, the protocol achieved satisfactory adherence and meaningful physiological improvements. Because this modality requires minimal equipment and space, it can be readily integrated into the daily routines of college students. Considering the improvements observed in physical fitness (CRF and balance), along with its potential to enhance sleep quality, campus-based running HIIT emerges as a low-cost, easily implementable strategy with meaningful potential to mitigate health risks associated with sedentary behavior among university students. From a student health perspective, university administrators might consider incorporating such accessible exercise protocols into broader campus health policies. For instance, integrating brief HIIT routines into university health-promotion systems, such as campus-based applications or digital platforms, could offer a scalable and sustainable approach to encourage wider student participation.

## Limitations

Several limitations should be noted. First, the six-week intervention period and relatively modest training dose may be insufficient to elicit detectable group-level changes in outcomes such as sleep quality or body composition, which typically require longer adaptation periods. Second, although the sample size met statistical requirements, the statistical power may still be limited for variables with smaller effect sizes, such as sleep outcomes. Third, the study did not monitor potential confounders such as electronic screen exposure or psychological stress. These behavioral and psychosocial factors might be associated with sleep in university students and may reduce the ability to isolate the independent effects of HIIT. Another methodological limitation worth noting is our reliance on subjective sleep questionnaires and estimated VO_2max_ through exercise testing protocols. While these methods are validated and practical for field research, future studies with access to advanced laboratory facilities could employ more precise measurement techniques, such as polysomnography for sleep monitoring and direct gas analysis for VO_2max_ assessment, potentially providing more comprehensive insights into physiological adaptations.

## Future research

Future research should first employ larger and more diverse samples to improve statistical power and enhance the generalizability of the findings. Furthermore, longer-term interventions aligned with academic semester schedules are needed to evaluate both cumulative and sustained training effects. In addition, future trials could be conducted on female university students to determine whether comparable adaptive changes occur across sexes. Furthermore, systematic monitoring of key confounding factors, such as screen exposure and psychological stress, is essential to better isolate the independent effects of HIIT. Finally, adopting more objective assessment methods, including polysomnography or accelerometry for sleep and activity monitoring and direct gas-analysis techniques for determining VO_2max_, will further improve the accuracy and validity of outcome measurements [87, 88].

## Conclusion

This study demonstrates that a six-week unsupervised running-based high-intensity interval training program conducted in a campus setting can effectively improve CRF in sedentary male college students and produce modest gains in balance performance. Notably, the program demonstrated good adherence and safety, with no reported adverse events. Although the group-by-time interaction for sleep quality did not reach statistical significance, the favorable trend observed within the intervention group suggests a potential beneficial effect of HIIT on sleep. Overall, this unsupervised running-based HIIT offers advantages such as low time cost, minimal facility requirements, and high feasibility, making it a practical option in resource-limited university environments. It may serve as an effective component of campus health-promotion strategies aimed at mitigating cardiorespiratory fitness and sleep problems associated with sedentary lifestyles among male students.

## Supplementary Information

Supplementary Material 1.

## Data Availability

The datasets generated and analyzed during the current study are available in the Figshare repository (DOI: https://doi.org/10.6084/m9.figshare.31861276) upon reasonable request.

## Abbreviations

BIA: Bioelectrical impedance analysis
BMI: Body mass index
CRF: Cardiorespiratory fitness
GEE: Generalized estimating equation
HIIT: High-intensity interval training
HR_max_: Maximal heart rate
IPAQ: International Physical Activity Questionnaire
IPAQ-SF: International Physical Activity Questionnaire–Short Form
PACER: Progressive Aerobic Cardiovascular Endurance Run
PSQI: Pittsburgh Sleep Quality Index
PAR-Q: Physical Activity Readiness Questionnaire
RCT: Randomized controlled trial
RPE: Rating of perceived exertion
SBRN: Sedentary Behaviour Research Network
VO_2max_: Maximal oxygen uptake
