# Supplementary material for: Effects of unsupervised campus HIIT on fitness and sleep in sedentary male students: a randomized controlled trial
Source: BMC Public Health. 2026 Apr 9;26:1635. doi: 10.1186/s12889-026-27310-7 (PMC13195952; doi:10.1186/s12889-026-27310-7)
Supplement: Supplementary file 1 — Supplementary Material 1. [file 12889_2026_27310_MOESM1_ESM.pdf]

Supplementary Material A CONSORT 2025 checklist of information to include when reporting a randomised trial

Section / Topic	No	CONSORT 2025 checklist item description	Reported on page no.
**Title and abstract**			
Title and structured abstract	1a	Identification as a randomised trial	Title (p.1)
	1b	Structured summary of the trial design, methods, results, and conclusions	Abstract (p.1-2)
**Open science**			
Trial registration	2	Name of trial registry, identifying number (with URL) and date of registration	p.2, 7
Protocol and statistical analysis plan	3	Where the trial protocol and statistical analysis plan can be accessed	Not available
Data sharing	4	Where and how the individual de-identified participant data (including data dictionary), statistical code and any other materials can be accessed	Data availability section (p.25)
Funding and conflicts of interest	5a	Sources of funding and other support (e.g., supply of drugs), and role of funders in the design, conduct, analysis and reporting of the trial	Funding (p.25)
	5b	Financial and other conflicts of interest of the manuscript authors	Competing interests (p.25)
**Introduction**			
Background and rationale	6	Scientific background and rationale	p.3-5
Objectives	7	Specific objectives related to benefits and harms	p.5-6
**Methods**			
Patient and public involvement	8	Details of patient or public involvement in the design, conduct and reporting of the trial	Not applicable
Trial design	9	Description of trial design including type of trial (e.g., parallel group, crossover), allocation ratio, and framework (e.g., superiority, equivalence, non-inferiority, exploratory)	p.7-8
Changes to trial protocol	10	Important changes to the trial after it commenced including any outcomes or analyses that were not prespecified, with reason	15
Trial setting	11	Settings (e.g., community, hospital) and locations (e.g., countries, sites) where the trial was conducted	p.12-13
Eligibility criteria	12a	Eligibility criteria for participants	p.6-7
	12b	If applicable, eligibility criteria for sites and for individuals delivering the interventions (e.g., surgeons, physiotherapists)	Not applicable

Intervention and comparator	13	Intervention and comparator with sufficient details to allow replication. If relevant, where additional materials describing the intervention and comparator (e.g., intervention manual) can be accessed	p.7-8
Outcomes	14	Pre-specified primary and secondary outcomes, including the specific measurement variable (e.g., systolic blood pressure), analysis metric (e.g., change from baseline, final value, time to event), method of aggregation (e.g., median, proportion), and time point for each outcome	p.8-11
Harms	15	How harms were defined and assessed (e.g., systematically, non-systematically)	p.11 + p.13
Sample size	16a	How sample size was determined, including all assumptions supporting the sample size calculation	p.6
	16b	Explanation of any interim analyses and stopping guidelines	Not applicable
Randomisation:			p.7
Sequence generation	17a	Who generated the random allocation sequence and the method used	p.7-8
	17b	Type of randomisation and details of any restriction (e.g., stratification, blocking and block size)	p.7
Allocation concealment mechanism	18	Mechanism used to implement the random allocation sequence (e.g., central computer/telephone; sequentially numbered, opaque, sealed containers), describing any steps to conceal the sequence until interventions were assigned	p.7
Implementation	19	Whether the personnel who enrolled and those who assigned participants to the interventions had access to the random allocation sequence	Not applicable
Blinding	20a	Who was blinded after assignment to interventions (e.g., participants, care providers, outcome assessors, data analysts)	p.7
	20b	If blinded, how blinding was achieved and description of the similarity of interventions	Not applicable
Statistical methods	21a	Statistical methods used to compare groups for primary and secondary outcomes, including harms	p.15
	21b	Definition of who is included in each analysis (e.g., all randomised participants), and in which group	p.15
	21c	How missing data were handled in the analysis	Not reported
	21d	Methods for any additional analyses (e.g., subgroup and sensitivity analyses), distinguishing prespecified from post-hoc	p.15
**Results**			
Participant flow, including flow diagram	22a	For each group, the numbers of participants who were randomly assigned, received intended intervention, and were analysed for the primary outcome	p.13-15
	22b	For each group, losses and exclusions after randomisation, together with reasons	p.13-15
Recruitment	23a	Dates defining the periods of recruitment and follow-up for outcomes of benefits and harms	p.15
	23b	If relevant, why the trial ended or was stopped	Not applicable
Intervention and comparator delivery	24a	Intervention and comparator as they were actually administered (e.g., where appropriate, who delivered the intervention/comparator, how participants adhered, whether they were delivered as intended [fidelity])	p.9-11

	24b	Concomitant care received during the trial for each group	P.16-20
Baseline data	25	A table showing baseline demographic and clinical characteristics for each group	Table 2 (p.16)
Numbers analysed, outcomes and estimation	26	For each primary and secondary outcome, by group:<br>● the number of participants included in the analysis<br>● the number of participants with available data at the outcome time point<br>● result for each group, and the estimated effect size and its precision (such as 95% confidence interval)<br>● for binary outcomes, presentation of both absolute and relative effect size	p.16–20
Harms	27	All harms or unintended events in each group	Not reported
Ancillary analyses	28	Any other analyses performed, including subgroup and sensitivity analyses, distinguishing pre-specified from post-hoc	p.15
**Discussion**			
Interpretation	29	Interpretation consistent with results, balancing benefits and harms, and considering other relevant evidence	p.20-23
Limitations	30	Trial limitations, addressing sources of potential bias, imprecision, generalisability, and, if relevant, multiplicity of analyses	p.23

Citation: Hopewell S, Chan AW, Collins GS, Hróbjartsson A, Moher D, Schulz KF, et al. CONSORT 2025 Statement: updated guideline for reporting randomised trials. BMJ. 2025; 388:e081123. https://dx.doi.org/10.1136/bmj-2024-081123.

© 2025 Hopewell et al. This is an Open Access article distributed under the terms of the Creative Commons Attribution License (https://creativecommons.org/licenses/by/4.0/), which permits unrestricted use, distribution, and reproduction in any medium, provided the original work is properly cited.

Supplementary Material B Intraclass correlation coefficients (ICCs) for outcome measures

Outcome	ICC[#]
Weight	0.995
Body fat	0.993
Body fat percentage	0.985
Muscle mass	0.955
Upper limb muscle	0.919
Lower limb muscle	0.955
BMI	0.995
Waist circumference	0.978
Hip circumference	0.944
Balance	0.863
Agility	0.478
Flexibility	0.931
VO_{2max}	0.854
Core endurance	0.828
Sleep quality	0.126
Physical activity	0.744

Units are as follows: weight (kg), body fat (kg), body fat percentage (%), muscle mass (kg), BMI (kg/m^2), circumference (cm), balance and core endurance (s), agility (s), flexibility (cm), VO_{2max} ($mL \cdot kg^{-1} \cdot min^{-1}$), and physical activity (MET-min/week).

[#]Based on pre- and post-test data of the control group.

Supplementary Material C Session-by-session attendance and intensity compliance

Week	Session Number	Attendance Rate	Intensity Compliance Rate
Week 1	Session 1	100% (28/28)	75% (21/28)
	Session 2	100% (28/28)	75% (21/28)
	Session 3	100% (28/28)	78.57% (22/28)
Week 2	Session 4	100% (28/28)	75% (21/28)
	Session 5	100% (28/28)	82.14% (23/28)
	Session 6	100% (28/28)	78.57% (22/28)
Week 3	Session 7	100% (28/28)	78.57% (22/28)
	Session 8	100% (28/28)	85.71% (24/28)
	Session 9	100% (28/28)	82.14% (23/28)
Week 4	Session 10	100% (28/28)	89.29% (25/28)
	Session 11	100% (28/28)	92.86% (26/28)
	Session 12	100% (28/28)	92.86% (26/28)
Week 5	Session 13	100% (28/28)	100% (28/28)
	Session 14	100% (28/28)	92.86% (26/28)
	Session 15	100% (28/28)	89.29% (25/28)
Week 6	Session 16	100% (28/28)	92.86% (26/28)
	Session 17	100% (28/28)	100% (28/28)
	Session 18	100% (28/28)	96.43% (27/28)
Overall	Total 1-18	100%	86.51%

*A session was considered "intensity-compliant" if the participant reached ≥ 80% HRmax in at least 8 out of the 10 high-intensity intervals

Supplementary Material D Sensitivity Analysis Results

Table 1 Effects of HIIT on physical fitness and sleep quality

Outcome	HIIT group (n = 30)	Control group (n = 29)	p	Group × Time		
				β (95% CI)*	p	ES#
**PACER**						
Pre	39.10 (13.64)	34.21 (13.78)	0.176 (t = 1.371)	−16.77 (−22.62, −10.92)	<.001	1.224
Post	56.77 (17.68)	33.10 (11.59)	<.001 (Z = −4.543)			
p	<.001 (t = −6.355)	0.760 (Z = −0.305)				
**VO_{2max}**						
Pre	30.47 (5.83)	28.96 (6.96)	0.368 (t = 0.907)	−6.21 (−8.30, −4.12)	<.001	0.969
Post	36.03 (5.97)	28.31 (5.91)	<.001 (t = 4.989)			
p	<.001 (t = −6.301)	0.316 (t = 1.022)				
**Balance**						
Pre	38.93 (24.86)	58.63 (54.23)	0.299 (Z = −1.039)	−21.69 (−38.83, −4.56)	0.013	0.517
Post	56.92 (38.96)	54.92 (55.61)	0.262 (Z = −1.122)			
p	0.004 (Z = −2.869)	0.716 (Z = 0.364)				
**Agility**						
Pre	14.86 (2.00)	15.33 (1.00)	0.422 (Z = −0.804)	−0.78 (−2.24, 0.68)	0.293	0.493
Post	14.71 (3.56)	14.41 (2.78)	0.324 (Z = −0.986)			
p	0.031 (Z = −2.152)	0.015 (Z = −2.437)				
**Flexibility**						
Pre	24.49 (11.19)	23.70 (10.18)	0.777 (t = 0.285)	−1.39 (−4.62, 1.85)	0.401	0.129
Post	26.22 (10.07)	24.04 (12.64)	0.466 (t = 0.734)			
p	0.253 (t = −1.166)	0.674 (t = −0.425)				
**Core endurance**						
Pre	119.60 (50.46)	104.60 (40.30)	0.213 (t = 1.258)	−11.10 (−28.61, 6.41)	0.214	0.243
Post	130.64 (63.83)	104.54 (45.56)	0.077 (t = 1.802)			
p	0.165 (t = −1.426)	0.838 (Z = −0.205)				
**Sleep**						
Pre	4.97 (2.34)	5.43 (2.44)	0.546 (t = −0.608)	0.87 (−0.37, 2.12)	0.170	0.368
Post	4.23 (1.81)	5.48 (1.75)	0.009 (t = −2.697)			
p	0.066 (t = 1.913)	0.793 (t = −0.265)				

Note: Values are presented as mean ± SD.
Units are as follows: PACER (laps), VO_{2max} ($mL \cdot kg^{-1} \cdot min^{-1}$), balance (s), agility (s), flexibility (cm) and, core endurance (s).
*Adjusted for age and pre-intervention protein intake.
#Effect sizes were reported as the absolute values of Cohen's d.

Table 2 Effects of HIIT on body composition and physical activity

Outcome	HIIT group (n = 30)	Control group (n = 29)	*p*	Group × Time		
				β (95% CI)*	*p*	*ES*#
**Weight**						
Pre	68.33 (8.55)	72.85 (14.02)	0.144 (t = −1.488)	−0.61 (−2.40, 1.82)	0.506	0.052
Post	68.84 (8.73)	72.75 (13.97)	0.205 (t = −1.285)			
p	0.570 (t = −0.574)	0.776 (Z = −0.285)				
**Body fat**						
Pre	14.26 (5.55)	17.19 (9.76)	0.422 (Z = −0.804)	−0.41 (−1.67, 0.84)	0.520	0.052
Post	14.26 (5.69)	16.77 (9.39)	0.940 (Z = −0.076)			
p	0.992 (t = 0.011)	0.053 (Z = −1.937)				
**Body fat percentage**						
Pre	0.20 (0.06)	0.22 (0.08)	0.213 (t = −1.259)	−0.01 (−0.01, 0.01)	0.685	0.141
Post	0.20 (0.06)	0.21 (0.08)	0.256 (t = −1.147)			
p	0.775 (t = 0.289)	0.046 (t = 2.089)				
**Muscle mass**						
Pre	49.99 (8.15)	52.65 (4.94)	0.137 (t = −1.509)	−0.60 (−2.20, 0.85)	0.416	0.089
Post	51.06 (7.95)	52.11 (5.25)	0.247 (t = −1.169)			
p	0.140 (t = −1.518)	0.095 (t = −1.729)				
**Upper limb muscle**						
Pre	4.85 (0.97)	4.99 (0.63)	0.522 (t = −0.645)	−0.04 (−0.26, 0.18)	0.702	0.053
Post	4.92 (0.96)	5.02 (0.72)	0.673 (t = −0.425)			
p	0.493 (t = −0.694)	0.245 (Z = −1.163)				
**Lower limb muscle**						
Pre	19.61 (2.78)	20.40 (2.15)	0.226 (t = −1.223)	−0.44 (−1.11, 0.24)	0.205	0.175
Post	20.13 (2.59)	20.48 (1.99)	0.557 (t = −0.590)			
p	0.130 (t = −1.560)	0.499 (t = −0.686)				
**BMI**						
Pre	22.49 (2.81)	23.65 (4.21)	0.219 (t = −1.244)	−0.19 (−0.74, 0.369)	0.498	0.054
Post	22.65 (2.99)	23.61 (4.18)	0.312 (t = −1.021)			
p	0.574 (t = −0.569)	0.673 (t = 0.427)				
**Waist circumference**						
Pre	79.22 (6.53)	81.43 (10.30)	0.398 (t = −0.851)	−0.28 (−2.39, 1.82)	0.791	0.032
Post	78.98 (7.65)	80.91 (10.73)	0.844 (Z = −0.197)			
p	0.817 (t = 0.233)	0.201 (Z = −1.278)				
**Hip circumference**						

Outcome	HIIT group (n = 30)	Control group (n = 29)	*p*	Group × Time		
				β* (95% CI)	***p***	***ES*#**
Pre	96.93 (6.53)	98.44 (7.66)	0.418 (t = −0.817)	1.45 (−0.55, 3.46)	0.155	0.204
Post	95.97 (5.27)	98.93 (7.56)	0.088 (t = −1.741)			
p	0.312 (t = 1.030)	0.223 (Z = −1.219)				
**Physical activity**						
Pre	2033.13 (2004.60)	2379.21 (1867.54)	0.363 (Z = −0.910)	−397.42 (−1280.87, 486.04)	0.378	0.205
Post	2299.48 (2020.57)	2248.14 (1683.51)	0.940 (Z = −0.076)			
p	0.199 (Z = −1.286)	0.591 (t = 0.544)				

Note: Values are presented as mean ± SD.

Units are as follows: weight (kg), body fat (kg), body fat percentage (%), muscle mass (kg), BMI (kg/m^2), circumference (cm) and physical activity (MET-min/week).

*Adjusted for age and pre-intervention protein intake.

#Effect sizes were reported as the absolute values of Cohen's d.
